# Personalized immunotherapy for non-small cell lung cancer through identification of tumor-specific mutations by next generation sequencing and adoptive transfer of tumor infiltrating lymphocytes that recognize neoantigens

**DOI:** 10.1186/2051-1426-3-S2-P20

**Published:** 2015-11-04

**Authors:** Ken-ichi Hanada, Christopher Chow, Raul Gil Hoyos, Jared J Gartner, Todd D Prickett, Robert Somerville, Katherine Hogan, Paul F Robbins, Steven A Rosenberg, James C Yang

**Affiliations:** 1 NCI, NIH, Bethesda, MD, USA; 2 Surgery Branch/National Cancer Institute / National Institutes of Health, Bethesda, MD, USA; 3 National Institutes of Health, Bethesda, MD, USA

## Background

Patients with metastatic melanoma can be successfully treated with adoptive transfer of tumor infiltrating lymphocytes (TIL). In 93 patients with over 5 year follow-up, the overall response rate was 56% and 20% achieved durable complete responses persisting in excess of 7 years. However, past attempts to apply TIL therapy to other solid cancers have not been successful and low frequency of tumor-specific T cells in other cancers has been suspected as a reason.

Recent progress in Next Generation Sequencing technology has enabled us to analyze genetic mutations in an individual patient's tumor and identify immune cells that are reactive to these mutation-encoded neoantigens. The potential of this therapeutic approach recently was illustrated in a patient with cholangiocarcinoma who experienced major tumor regression when given such T cells. NSCLC is a cancer with a high number of genetic mutations and a recent report suggests that clinical response of NSCLC to anti-PD1 antibody therapy is positively associated with the number of genetic mutations.

## Results

To identify neoantigen-reactive T cells from NSCLC and utilize them for adoptive therapy, we initiated clinical protocol (NCT02133196). Initial Whole Exome Sequence analysis of freshly resected metastases from four patients with NSCLC showed between 150 and 1500 non-synonymous mutations that could be confirmed by RNAseq. TIL cultures from these tumors tend to be initially dominated by CD3^-^, CD56^+^ cells, gradually converting to CD3^+^CD4^+^ dominant, CD3^+^CD8^+^ dominant, or a mixture of both at approximately 3 weeks. In all the cases, we were able to find CD4^+^ and/or CD8^+^ T cell populations that were reactive to one or more autochthonous neoantigens as confirmed by IFN-γ ELISPOT and FACS analysis based on 4-1BB and/or OX-40 up-regulation. In multiple TIL cultures, more than 50% of the cells were reactive to a neoantigen and in one case, single mutation in NPM1 (nucleophosmin) yielded both CD4^+^ and CD8^+^ T cell reactivity. These T cells can be grown to large numbers *in vitro* for patient adoptive transfer and these studies are underway.

